# MicroRNA and Target Protein Patterns Reveal Physiopathological Features of Glioma Subtypes

**DOI:** 10.1371/journal.pone.0020600

**Published:** 2011-05-31

**Authors:** Elodie Lages, Audrey Guttin, Michèle El Atifi, Claire Ramus, Hélène Ipas, Isabelle Dupré, Delphine Rolland, Caroline Salon, Catherine Godfraind, Florence deFraipont, Mehdi Dhobb, Laurent Pelletier, Didier Wion, Emmanuel Gay, François Berger, Jean-Paul Issartel

**Affiliations:** 1 Team7 Nanomedicine and Brain, INSERM U836, Grenoble, France; 2 Institut des Neurosciences, Université Joseph Fourier, Grenoble, France; 3 Clinical Transcriptomics and Proteomics Platform, Centre Hospitalier Universitaire and Grenoble Institut des Neurosciences, Grenoble, France; 4 Laboratoire d’Hématologie Cellulaire et Moléculaire, Département d’Hématologie, Onco-Génétique et Immunologie, Centre Hospitalier Universitaire, Grenoble, France; 5 Department of Pathology, Centre Hospitalier Universitaire, Grenoble, France; 6 Laboratory of Pathology, Cliniques Universitaires St-Luc, Université Catholique de Louvain, Brussels, Belgium; 7 Unité Médicale de Biochimie des Cancers et Biothérapies, Département de Biochimie, Toxicologie et Pharmacologie, Centre Hospitalier Universitaire, INSERM U823, Université Joseph Fourier, Grenoble, France; 8 Department of Neurosurgery, Centre Hospitalier Universitaire, Grenoble, France; 9 CNRS, Grenoble, France; City of Hope National Medical Center and Beckman Research Institute, United States of America

## Abstract

Gliomas such as oligodendrogliomas (ODG) and glioblastomas (GBM) are brain tumours with different clinical outcomes. Histology-based classification of these tumour types is often difficult. Therefore the first aim of this study was to gain microRNA data that can be used as reliable signatures of oligodendrogliomas and glioblastomas. We investigated the levels of 282 microRNAs using membrane-array hybridisation and real-time PCR in ODG, GBM and control brain tissues. In comparison to these control tissues, 26 deregulated microRNAs were identified in tumours and the tissue levels of seven microRNAs (miR-21, miR-128, miR-132, miR-134, miR-155, miR-210 and miR-409-5p) appropriately discriminated oligodendrogliomas from glioblastomas. Genomic, epigenomic and host gene expression studies were conducted to investigate the mechanisms involved in these deregulations. Another aim of this study was to better understand glioma physiopathology looking for targets of deregulated microRNAs. We discovered that some targets of these microRNAs such as STAT3, PTBP1 or SIRT1 are differentially expressed in gliomas consistent with deregulation of microRNA expression. Moreover, MDH1, the target of several deregulated microRNAs, is repressed in glioblastomas, making an intramitochondrial-NAD reduction mediated by the mitochondrial aspartate-malate shuttle unlikely. Understanding the connections between microRNAs and bioenergetic pathways in gliomas may lead to identification of novel therapeutic targets.

## Introduction

Gliomas are brain tumours typed as oligodendrogliomas, astrocytomas, high-grade astrocytomas (glioblastomas) and several other subtypes, which are an important cause of mortality in adults and children. The original cell type(s) of these tumours is still uncertain and the molecular determinants of their aggressiveness have been the subject of numerous investigations [1]. The incorporation of molecular information would help the pathologist to discriminate between these tumour types, which show variable pathology and are known for their heterogeneity. Molecular analyses are expected to reveal appropriate markers of gliomas for typing and prognosis. In particular, genome-wide gene expression profiles were studied to stratify gliomas and to define the intrinsic features of the subtypes [2], [3]. Other attempts have focused on other transcriptomic markers and were based on analyses of expression levels of microRNAs in tumour tissues (for review [4]).

MicroRNAs (miRNAs) are short ∼22 nucleotide non-coding single-stranded RNAs that negatively regulate gene expression by binding to target mRNAs and, although less frequent, can stimulate translation of some mRNAs [4].

To date, close to 1100 different human miRNAs are referenced in the miRBase (release 16; sept 2010). They play a crucial role in the regulation of processes such as development, differentiation, cell proliferation and apoptosis, with many of these processes often altered in tumours (for review [5]). Moreover, miRNAs may be transferred between glioma cells and adjacent cells through gap junctions and induce targeted inhibition of protein expression in the acceptor cells [6] and they also participate in cell to cell signalling via an exosomal-mediated transfer between cells [7].

In this report, we measured the expression levels of 282 different miRNAs in tissues from oligodendrogliomas and glioblastomas that were classified by pathology analyses and on the basis of their molecular subtyping according to Li , *et al.* [3]. Specific miRNA patterns for these two different types of glioma were evidenced and we addressed two main questions: why are these miRNAs deregulated in this tumour context? what are the impacts of these deregulations on cellular pathways?

## Materials and Methods

The study was approved by the Biological Resource Center Ethics Review Board 38043 Hospital of Grenoble. Written consent was obtained from each patient or family.

### Biological samples

Glial tumour samples (12 oligodendrogliomas and 12 glioblastomas) and 4 control brain tissues were obtained at the time of surgery from patients in Grenoble University Hospital. Samples were immediately frozen at −80°C. Tissues came from newly diagnosed tumours that were typed according to the latest WHO classification (2007) by two neuropathologists (C. Salon and C. Godfraind). In addition, expression data of 54 Affymetrix Genechip probe sets were used for typing the tumour samples according to the gene expression signature-based classification proposed by Li , *et al*. [3]. Oligodendrogliomas (ODG) and glioblastomas (GBM) in this study were sorted into the O group and G group, respectively, with a confidence value higher than 85% compared to the classifiers of Li , *et al.* [3]). Tumour samples were also characterised by the gene expression signature-based classification proposed by Phillips , *et al*. [2], sequencing of a PCR fragment of the *IDH1* gene to seek codon 132 mutations [1], [8], detection of the 1p19q LOH [1], as well as other clinical features (supplementary Table S1).

Human glioblastoma cells (U87 or low passage cell lines) were cultured in standard DMEM medium supplemented with 10% fetal bovine serum. The cells were submitted to different O_2_ concentrations (0.3%, 3% and 20%) for 24 h before harvesting.

### RNA extraction

RNAs were extracted from cells or tissue samples using the mirVana™ miRNA isolation kit (Ambion). Low-molecular-weight (LMW) RNAs (<250 bp) were purified and separated from high-molecular-weight (HMW) RNAs (>250 bp). The LMW and HMW quality was checked with the RNA Nano 6000 kit on Bioanalyzer 2100 (Agilent Technologies).

### Membrane-array hybridisation and real-time PCR

Hybridisation was analysed using nylon membranes on which oligonucleotide probes complementary to mature miRNA sequences (Ambion) were spotted in duplicate. LMW RNAs (2 µg) were labelled by radioactive poly(A) tailing with 2 µCi of ATP (a^33^P) (PerkinElmer Life) and 2 U of poly(A)polymerase (Ambion). Labelled RNAs were hybridised to the membranes for 2 days at 50°C; then the arrays were washed twice with 5X SSC, 0.5% SDS, and exposed on a phosphor screen for 48 h and scanned with a BAS 5000 Phosphor scanner (Fujifilm Raytest). The signals were quantified with Array Gauge 4.0 software (Fujifilm) and normalised by background subtraction and global normalisation.

Real-time PCR analyses of LMW RNAs were based on mature miRNA-specific Taqman^®^ MicroRNA assays (Applied Biosystems). Ct measurements were taken on a Stratagene Mx3005p system (Agilent Technologies). RNU24 small nucleolar RNA was used as an internal normalization reference. The calculations were made using the Relative Expression Software Tool (REST) (http://www1.qiagen.com/products/rest2009software.aspx).

### mRNA profiling

Gene expression profiles were analysed on GeneChip^®^ Human Genome U133 Plus 2.0 (Affymetrix) corresponding to 47,401 unique transcripts. Microarrays were used in accordance with the manufacturer's protocol. In brief, 5 µg of HMW RNA was reverse transcribed to single-stranded cDNA and after second-strand cDNA synthesis, biotin-labelled antisense cRNA was generated by *in vitro* transcription. The cRNA preparation was fragmented and hybridised to the oligonucleotide microarray and then washed, stained and scanned. Raw expression data were analysed using GeneChip Operating software (GCOS) version 1.2 (Affymetrix) and Robust Multi-array Average (RMA) analysis.

### CpG mapping and DNA methylation analysis

CpG islands were sought in the 2000-bp region upstream of the miRNA gene transcription start sites previously described by Marson , *et al.* [9] using Methyl Primer Express v1.0 software (Applied Biosystems) or as described for miR-124-2, miR-127 and miR-128 [10]–[12].

Genomic DNA extracted using standard protocols (Qiagen) was treated with sodium bisulfite using the EZ DNA methylation-Gold kit according to the manufacturer’s protocol (Zymo Research). Amplification by PCR and pyrosequencing (primer list, supplementary Table S2) were performed and the methylation rate at each CpG position was calculated (PyroMark ID Biotage QIAGEN).

### Western blot analysis

Tissues were lysed using Promega lysis buffer including protease inhibitors. Western blots were performed with PVDF membranes. Antibodies were from Santa Cruz Biotechnology (BCL2, SNAP25, THBS1, MDM2, SIRT1, anti-mouse and anti-rabbit horseradish peroxidase-conjugated secondary antibodies); Abcam (CD44, MDH1, STAT3, Y705P-STAT3, CCPG1, PTBP1) or Neomarkers (beta-actin). Signals were detected by chemiluminescent detection (ECL, Amersham Biosciences) and protein amounts were normalised to the beta actin signals. Images were processed with ImageJ software (http://rsbweb.nih.gov/ij/).

### Data analysis, statistics, database

The results are shown as the mean of at least triplicate individual measurements. Statistical significance was assessed using the two-tailed *t*-test as well as ANOVA as appropriate. *P*-values less than 0.05 were considered statistically significant. We used a threefold expression difference as a cutoff level. We used the Sanger database (http://www.ebi.ac.uk/enright-srv/microcosm/htdocs/targets/v5/) to identify relevant miRNA targets. Pathway rationalisation was performed with Ingenuity Pathway Analysis.

## Results

### miRNA contents in glioma tissues

Brain tumour and control tissues were analysed for miRNA contents using two different techniques, hybridisation onto membrane arrays and real-time PCR. A total of 282 different miRNAs were measured in the samples (supplementary Table S3).

The results were expressed as the ratios, for every single miRNA, of their average amounts measured in gliomas (glioblastoma [GBM] or oligodendroglioma [ODG]) to their amounts in control samples. MiRNAs with ratios higher than 3 or lower than 0.33 (either GBM to control or ODG to control) obtained by any of the two methods were considered glioma markers and retained for further analysis.

A total of 26 different miRNAs fulfilled these criteria and are listed in Table 1. Eighteen of these 26 miRNAs were detected by both methods. Most of the time, over- or under-expression of miRNAs in the gliomas compared to the control tissue, respectively, was consistently detected with both techniques. We assumed that discrepancies were related to the fact that the dynamic range of the recorded hybridisation signals seemed to be shrunken in comparison to that of the PCR measurements.

**Table 1:** Deregulated miRNAs in gliomas versus control brain tissue.

	Real-time PCR			Membrane-array		
miRNA	GBM/N	ODG/N	GBM/ ODG	GBM/N	ODG/N	GBM/ ODG
miR-21	**87.8** †	**9.0**	**9.8**	**4.8**	1.3	**3.7**
miR-155	**34.5**	**3.1**	**11.2**	**4.8**	0.6	**8.4**
let-7f	**23.2**	**25.8**	0.9	**10.6**	**5.2**	2.0
let-7a	**19.2**	**20.5**	0.9	**4.1**	**4**	1.0
miR-17	**12.9**	**17.9**	0.7	*ND*	*ND*	*ND*
miR-16	**11.4**	**5.0**	2.3	**4.5**	2.7	1.7
miR-26b	**9.0**	**7.1**	1.3	*ND*	*ND*	*ND*
miR-374a	**7.8**	**7.4**	1.0	*ND*	*ND*	*ND*
miR-126	4.8*	2.8	1.7	*ND*	*ND*	*ND*
let-7d	**4.2**	**3.0**	1.4	1.6	2.3	0.7
miR-20a	**3.6**	**5.0**	0.7	*ND*	*ND*	*ND*
miR-15b	**3.3**	**3.4**	1.0	**4.3**	1.2	**3.7**
let-7b	**3.3**	**3.4**	1.0	1.3	1.8	0.7
miR-9	2.1	**5.7**	0.4	2.1	**3.5**	0.6
miR-210	2.3	*0.29*	**8.1**	**5.6**	0.5	**10.7**
miR-409-5p	*0.32* ‡	*0.07*	**4.6**	*ND*	*ND*	*ND*
miR-132	0.28*	*0.09*	**3.2**	0.5	*0.28*	1.8
miR-134	*0.20*	*0.04*	**5.8**	2.7	*0.19*	**14.2**
miR-149	0.18*	*0.10*	1.8	*ND*	*ND*	*ND*
miR-128	*0.12*	0.46	*0.26*	*0.29*	*0.33*	0.9
miR-7	*0.11*	*0.09*	1.2	*0.15*	*0.15*	1.0
miR-330-3p	*0.08*	*0.05*	1.7	*0.25*	*0.32*	0.8
miR-139-5p	*0.07*	*0.09*	0.8	*0.13*	*0.08*	1.6
miR-339-5p	0.07*	*0.11*	0.6	*ND*	*ND*	*ND*
miR-127-3p	*0.06*	*0.03*	2.0	0.7	*0.06*	**11.9**
miR-124	*0.00*	*0.00*	1.0	*0.2*	*0.11*	1.8

miRNAs were assayed by real-time PCR and hybridisation in glioblastomas (GBM), oligodendrogliomas (ODG) and control brain tissues (N). miRNA ratios between the different tissues are reported as indicated. Statistical significance was assessed using ANOVA. All *P*-values were lower than 0.05 (and between 0.05 and 0.2 when indicated by *).

†ratios above 3 are in boldface.

‡ratios below 0.33 are in italics.

ND: not detected.

**Figure 1:**
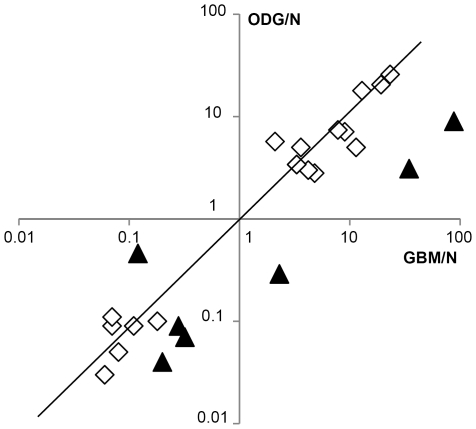
Deregulated miRNAs in gliomas. Correlation between miRNA expression in ODG versus miRNA expression in GBM. Data were obtained by real-time PCR. ODG/N and GBM/N miRNA ratios are expressed in log_10_(ratio value). Triangles: miRNAs with a GBM/ODG ratio higher than 3 or lower than 0.33. Diagon illustrates identical expression of miRNAs in glioblastomas and oligodendrogliomas.

Fourteen miRNAs had high glioma to control ratios and 11 had low ratios. Only miR-210 was found in higher amounts in GBM but in lower amounts in ODG than in control tissue. The results obtained by real-time PCR were plotted on a graph that displays the ODG to control tissue miRNA ratios versus the GBM to control tissue miRNA ratios (Fig. 1). This shows that there are obviously two common sets of miRNAs that are either over-expressed or under-expressed in gliomas in comparison to the control brain tissue. In addition, six miRNAs are over-expressed in GBM compared to ODG (GBM to ODG miRNA ratios above 3 in miR-21, miR-132, miR-134, miR-155, miR-210 and miR-409-5p). On the contrary, a higher level of expression of miR-128 was found in ODG than in GBM (Table 1).

### Coding region structures and miRNA expression

Alterations of the genome structure such as losses of heterozygosity, gene amplifications or changes in the miRNA sequences (SNPs or mutations that can interfere with the assays) may lead to variations of the total amount of assayed miRNAs. Genomic analyses were carried out to assess the copy number of the miRNA loci in the tissue samples by PCR (primers in supplementary Table S4). We specifically focused our attention on the miRNAs with low levels in gliomas (miR-7, miR-124, miR-127-3p, miR-128, miR-134, miR-139-5p, miR-149, miR-339-5p and miR-409-5p) and on three over-expressed miRNAs (miR-9, miR-21 and miR-155). For miR-128 which is encoded by two different loci and miR-7, miR-9 and miR-124 by three different loci, the copy numbers of all the loci were analysed. However, we were unable to amplify the miR-124-3 locus.

No deletion or gene amplification was found in the tumour tissues for the studied coding regions (supplementary Fig. S1). Using the Affymetrix SNP6.0 arrays, we noted that a 7p22.3 fragment (bp 52,899 to 1,466,894), which contains the miR-339 coding region, was present in triplicate copies in GBM. All PCR fragments were sequenced and proved free of mutation.

### Expression levels of the miRNA hosting genes

In a number of cases, miRNAs are hosted in the structure of coding or non-coding transcribed genes and some of them are co-expressed with their host genes in normal tissues [13]. Any correlation between host-gene transcript levels and miRNA amounts may be interpreted as a first clue regarding the mechanism of miRNA expression control. From Affymetrix hybridisation experiments we analysed the expression levels of 17 genes corresponding to 15 different mature miRNAs (Table 2).

**Table 2:** miRNA host gene expression in gliomas.

				Expression level			
				GBM/N		ODG/N	
	Host genes§			Host genes	micro RNAs	Host genes	micro RNAs
miRNA loci‡	Symbol	Name	Gene location				
**Coregulated hostgene and miRNA**							
miR-128-1	R3HDM1	R3H domain containing 1	2q21.3	*0.2* *	*0.1*	*0.3*	0.5
miR-128-2	ARPP-21	cyclic AMP-regulated phosphoprotein 21 kD	3p22.3	*0.1*	*0.1*	0.7	0.5
miR-139	PDE2A	phosphodiesterase 2A. cGMP-stimulated	11q13.4	*0.1*	*0.1*	*0.3*	*0.1*
miR-7-3	C19orf30	chromosome 19 open reading frame 30	19p13.3	*0.1*	*0.2*	*0.2*	0.1
**Poorly coregulated hostgene and miRNA**							
miR-155	MIR155HG	MIR155 host gene (non-protein coding)	21q21.3	**5.9** †	**34.5**	0.7	**3.1**
miR-15b	SMC4	structural maintenance of chromosome 4	3q26.1	**4.8**	**3.3**	1.8	**3.4**
miR-16-2	SMC4		3q26.1	**4.8**	**11.4**	1.8	**5.0**
miR-149	GPC1	glypican 1	2q37.3	2.8	*0.2*	0.9	*0.1*
miR-17	MIR17HG	MIR17 host gene (non-protein coding)	13q31.3	1.4	**12.9**	2.9	**17.9**
miR-20a	MIR17HG	MIR17 host gene (non-protein coding)	13q31.3	1.4	**3.6**	2.9	**5**
miR-26b	CTDSP1	CTD (carboxy-terminal domain, RNA polymerase II, polypeptide A) small phosphatase 1	2q35	1.4	**9.0**	1.6	**7.1**
miR-339	C7orf50	chromosome 7 open reading frame 50	7p22.3	1.2	*0.1*	0.9	*0.1*
miR-9-2	LOC645323		5q14.3	1.1	2.1	**3.0**	**5.7**
miR-16-1	DLEU2	deleted in lymphocytic leukemia 2 (non-protein coding)	13q14.2	1.0	**11.4**	0.9	**5.0**
miR-7-1	HNRNPK	heterogeneous nuclear ribonucleoprotein K	9q21.32	1.0	*0.2*	1.0	*0.1*
let-7f-2	HUWE1	HECT, UBA and WWE domain containing 1	Xp11.22	0.8	**23.2**	0.8	**25.8**
miR-126	EGFL7	EGF-like-domain. multiple 7	9q34.3	0.7	**4.8**	0.6	2.8
miR-330	EML2	echinoderm microtubule associated protein like 2	19q13.32	0.7	*0.1*	0.6	*0.1*
miR-9-1	C1orf61	chromosome 1 open reading frame 61	1q22	0.5	2.1	1.7	**5.7**

Host gene expression levels in gliomas were assessed by Affymetrix genechip hybridisation and normalized to the expression levels in control brain tissue. The expression levels of the mature miRNAs measured by real-time PCR (Table 1) are reported.

*ratios below 0.33 are in italics.

†ratios above 3 are in boldface.

‡miRNA loci corresponding to miRNAs that showed coexpression with their host genes in normal tissue as reported [13] are underlined.

§The 17 reported genes and transcribed regions host 19 different miRNA loci that code for 15 mature miRNAs.

For few miRNAs, we noted an apparent correlation between the changes in expression levels of the host genes and the respective changes of the mature miRNA amounts in gliomas. The genes that host two mature miRNAs, present in very low amounts in gliomas (miR-128 with two gene isoforms and miR-139-5p), are down-regulated in gliomas. The expression level of at least one (C19orf30) of the three isoforms that encode miR-7 (miR-7 is expressed at low levels in gliomas) was severely down-regulated in gliomas. The transcribed MIR155HG gene, which hosts the over-expressed miR-155 and the SMC4 gene, which hosts both the miR-15b and miR-16 were found over-expressed in GBM but not in ODG. In all the other cases host-gene expression was found to be deregulated in a way that is inconsistent with the variation of the expression levels of the hosted miRNAs in the tumours.

### Regulation of the expression levels of miRNA by epigenetic modifications

**Figure 2:**
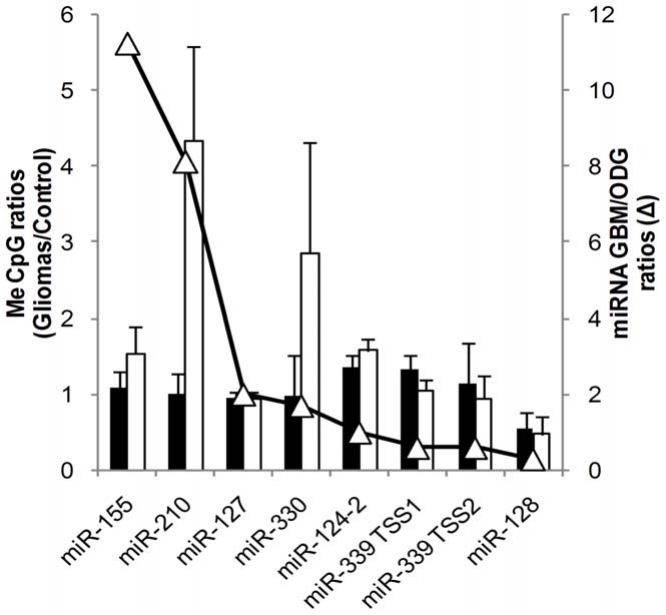
Methylation levels of CpG islands located upstream of the transcriptional starts of miRNA coding regions. Promoter regions of seven different miRNA genes were analyzed for the presence of CpG islands and for their methylation level. DNA fragments amplified from CpG islands were analyzed by pyrosequencing as described in Materials and Methods. Two different CpG islands were investigated for miR-339 coding region upstream transcription start sites, TSS1 and TSS2. Methylation levels are reported as ratios between gliomas (GBM or ODG) versus control brain tissue (left y-axis). Black bars: GBM/N ratios; white bars: ODG/N ratios. GBM to ODG miRNA expression levels are reported in open triangles (right y-axis).

Expression levels of miRNAs can also be controlled by epigenetic changes such as methylations of CpG islands in promoters. We screened the genomic sequences for the presence of CpG islands in the 2-kbp region upstream of the transcription start sites and analysed by pyrosequencing eight CpG islands upstream of the genes that encode seven different miRNAs (Fig. 2).

In GBM the levels of methylation of gene promoter regions was nearly identical to the levels in control tissues. On the contrary, the proportion of methylated CpGs in promoters of miR-210 and miR-330 was found significantly higher in ODG than in GBM and control tissue (57.7; 14.6 and 14.4 for miR-210, and 65.3; 22.6 and 26.6 for miR-330 in ODG, GBM and control tissue, respectively).

### Effect of hypoxia on miRNA gene expression

Since it is known that hypoxia can settle in gliomas, we checked whether extrinsic parameters such as oxygen concentration could affect the expression level of some miRNAs in tumour cells cultured *in vitro*. U87 glioblastoma cell cultures were subjected to different oxygen concentrations (20%, 3% and 0.3%) and the cellular miRNA contents were assessed. The expression of miR-15b, miR-16, miR-17, miR-20a, miR-210, let-7a, let-7b, let-7d and let-7f was found to be increased by hypoxia (the ratios miRNA levels at 0.3% O_2_ / miRNA level at 20% O_2_ ranged between 1.5 and 2.9). Oxygen concentration-dependent deregulation of the expression of miR-210 is shown in supplementary Fig. S2. On the contrary, for example, miR-100 expression was found to be unaffected by low oxygen concentration, suggesting that the expression of only a restricted set of miRNAs is regulated by the oxygen concentration. As a control, in the U87 glioma cells we also measured the overexpression of the CA9 (carbonic anhydrase IX), HIG2 (hypoxia-inducible gene-2) and LOX (lysyl oxidase) genes that have been reported to be overexpressed by hypoxia [14]. This confirmed that the U87 cells duly responded to our experimentally imposed oxygen deprivation.

### Target analysis

**Figure 3:**
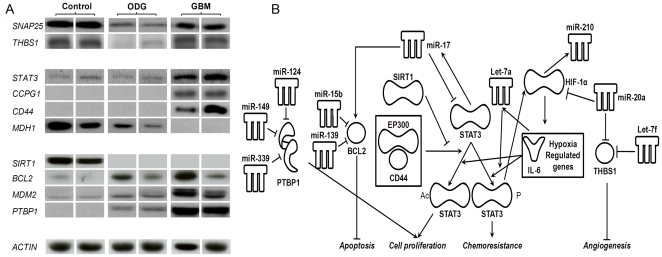
Immunodetection of target proteins and synopsis of their roles in cellular pathways and interactions with miRNAs. *A,* tissular lysates from control brain samples, ODGs and GBMs were analyzed by western blot with antibodies directed against the proteins indicated on the left. Equal quantities of proteins loaded on the gel were ascertained by immunodetection of beta-actin. A full length blot for detection of MDH1 is presented in supplementary Fig. S3. *B,* network with miRNAs and proteins. Protein names: see Table 3; EP300: E1A binding protein p300; STAT3 Ac: acetylated form of STAT3; IL6: interleukin 6; HIF-1α: hypoxia inducible factor-1alpha.

Several targets of the 26 glioma-specific miRNAs were selected and nine of them were studied because the amounts of the target proteins in tumour or control brain tissue lysates were found at a detectable level when assessed using Western blot (Fig. 3A). Detection of CD44 was also included because it has been found to be involved in miR-21 production [15] and in STAT3 activation [16].

**Table 3:** Expression levels of immunodetected proteins and their encoding mRNAs.

						Deregulated miRNAs targeting protein-coding mRNAs	
		GBM/N		ODG/N		Upregulated	Downregulated
		mRNA	Protein	mRNA	Protein		
**Moderate correlation between protein amounts and mRNA levels**							
SNAP25	synaptosomal-associated protein 25kDa	*0.0* *	0.6	*0.1*	*0.2*	miR-16	
STAT3	signal transducer and activator of transcription 3	2.8	**5.0**	1.0	0.9	miR-17, miR-20a	
CD44	CD44 molecule (Indian blood group)	**18.0** †	**∞**	1.8	1‡		
**Poor correlation between protein amounts and mRNA levels**							
MDH1	malate dehydrogenase 1, NAD (soluble)	*0.3*	0	*0.3*	*0.3*	miR-15b, miR-16, miR-26b, miR126	
SIRT1	sirtuin 1	0.5	0	1.5	0		miR-132
CCPG1	cell cycle progression 1	0.9	**∞**	1.0	1‡	miR-21, miR-155, miR-374a	miR-139-5p
BCL2	B-cell CLL/lymphoma 2	0.7	**3.5**	0.9	**3.3**	miR-15b, miR-17	miR-139-5p
MDM2	Mdm2 p53 binding protein homolog (mouse)	1.7	**8.3**	0.8	**3.0**	let-7b, let-7f	miR-339-5p
PTBP1	polypyrimidine tract binding protein 1	**3.2**	**∞**	1.1	**∞**		miR-124, miR-339-5p, miR-149
THBS1	thrombospondin 1	**3.6**	1.1	0.5	*0.2*	miR-21	

mRNA expression levels (from Affymetrix genechip hybridisation) and protein levels (from Western blots; Fig. 3) are given as the ratios between gliomas and control tissue. Up-regulated or down-regulated potential targeting miRNAs are indicated.

*ratios below 0.33 are in italics.

†ratios above 3 are in boldface.

‡undetectable levels in control and ODG.

∞undetectable levels in control samples.

Low amounts of SNAP25 and THBS1 were noted in ODG. High levels of STAT3, CCPG1 and CD44 but an undetectable amount of MDH1 were found in GBM (supplementary Fig. S3). The level of SIRT1 was low, while high amounts of BCL2, MDM2 and PTBP1 were noted in both types of tumours (Fig. 3A and Table 3). In addition, we also detected the Y705 phosphorylated form of STAT3 and found increased amounts of this phosphorylated form of STAT3 in GBM in comparison to non-tumour tissue and ODG (supplementary Fig. S4).

The targets can then be classified into two main groups (Table 3). One group corresponds to proteins that show changes in their tissue concentrations that grossly correlate with the mRNA levels (SNAP25, STAT3 and CD44). In the second group, the modification of the protein levels between tumours and control tissue poorly correlates with the mRNA level changes. For example, lower amounts than expected regarding mRNA levels are noted for MDH1 in GBM and SIRT1 in gliomas; or the protein concentration was increased in glioma tissues, although the mRNA was only slightly modified (typically CCPG1, BCL2, MDM2 and PTBP1). In this group, a modified concentration of specific miRNAs may be one of the possible events that controls the levels of proteins encoded by these genes.

## Discussion

### miRNAs for molecular phenotyping of gliomas

This analysis focused on an original comparison of two distinct groups of gliomas and was performed by two different technical and complementary approaches. With the choice of a cut-off set to ≥3-fold differential expression of the miRNAs between the samples, we were able to identify 26 miRNAs that appeared to be deregulated in glioma samples in comparison to control brain samples. Strikingly, the expression levels of most of the miRNAs identified were found to be very similar in the two types of tumour sample (Fig. 1). This suggests that a number of pathways for the regulation of the expression of several miRNAs are shared by ODG and GBM. Nevertheless, we found six miRNAs that showed more than three times the expression in the GBM group than in the ODG group of tumours. On the contrary, only one miRNA appeared more than three times overexpressed in ODG than in GBM. Accordingly, a set of seven markers of interest are described – miR-21, miR-128, miR-132, miR-134, miR-155, miR-210 and miR-409-5p – which clearly distinguishes the two groups of tumours.

As a consequence, this study has several implications for the pathology gliomas. A clear distinction between glioblastomas and low-grade tumours can be made feasible by molecular analyses based not only on mRNAs (as described for example in [2], [3]) but also on miARN measurements (supplementary Table S1). In the latter case, a small number of miRNA markers might be sufficient to distinguish GBM and ODG. This may indicate that appropriate RT-PCR assays or *in situ* hybridization using miRNA-targeting probes, performed in conjunction with conventional assays, may be of value in identification of glioma subtypes. Nevertheless, the specific miRNAs identified in this study are not yet sufficient alone to allow the distinction between low-grade tumour subtypes that are distinguished by the 1p and 19q LOH status.

miRNA contents in gliomas have already been analysed by several laboratories, but most of these analyses were done on astrocytomas and/or GBM, and few data have been reported on ODG (supplementary Table S5). Large-scale analyses that focus on glioma miRNA content and comparison between GBM and ODG are not sufficiently documented. In this study, we identified six miRNAs that, to the best of our knowledge, have never been reported as deregulated in gliomas in comparison to control brain tissue (let-7a, let-7b, let-7f and miR-374a, which are over-expressed in gliomas; miR-339-5p and miR-409-5p, which are under-expressed in gliomas). For 14 miRNAs in GBM, there is a consensus between our data and those reported in the literature (supplementary Table S5). Six miRNAs (miR-15b, miR-17, miR 20a, miR 21, miR 155 and miR 210) were found to be up-regulated, while eight others (miR-7, miR-127-3p, miR-128, miR-132, miR-134, miR-139-5p, miR-149 and miR-330-3p) were generally measured in low amounts in tumours. There is broad consensus, for example, in support of the over-expression of miR-21 and the under-expression of miR-7, miR-128 and miR-139-5p in gliomas. However, several discrepancies in all the published data can be found for let-7d, miR-9, miR-16, miR-26b, miR-124 and miR-126 (supplementary Table S5). Divergent results may be related to the heterogeneous nature of the tumour samples themselves used in the studies (tumour tissues or cultured cell lines), and most probably to the methodological approaches. In the present study, miRNAs were measured using two different methods. However, while the real-time PCR measurements strictly assay only one mature form of every single miRNA, hybridisation arrays may detect undetermined additional forms of miRNA with longer or end-frayed sequences in comparison to the mature miRNA form. In addition, using different RNAs to standardise the measurements in the different studies (let 7a or RNU6B or RNU24) might have a considerable influence on the final results.

Variations in the amounts of miRNA measured may indicate true tissue-dependent deregulations of the miRNA gene expression levels. However, other molecular events may interfere with the miRNA measurements and have adverse effects on these conclusions. Alterations in the mature miRNA sequences (mutations or SNPs), a potentially very critical problem in terms of reliability of the hybridisation or PCR-based assays of such small RNA components, may lead to apparently reduced amounts of detectable miRNAs. Variations may also be related to modifications of the copy number of miRNA genes, the activity of their corresponding promoters, or the post-transcriptional processing of the miRNAs. Finally, the miRNA expression levels may be dependent on the cellular type composition of the tumour samples themselves.

### Gene structure and copy number modifications inadequately explain the expression level of miRNA in gliomas

The copy numbers of the miR-7-1, miR-7-2, miR-7-3, miR-124-1, miR-124-2, miR-127, miR-128-1, miR-128-2, mirR134, miR-139, miR-149 and miR-409 genomic loci were found to be unaffected in the glioma samples and the sequences of all these miRNA genomic loci were found to be identical to the reference sequences (Supplementary Fig. S1). Therefore, it can be concluded that in comparison to the control brain samples, reduced expression of the corresponding miRNAs in tumour samples does not stem from gene deletions, mutations or SNPs. Similarly, no gene copy number modifications seem to be responsible for the high expression of mature miR-9, miR-21 and miR-155. Consequently, for miR-21, miR-128, miR-134, miR-155 and miR-409-5p, which are differentially expressed in GBM and ODG, no change in gene copy number was found to support the substantial difference in expression levels in the two different types of tumour.

However, we found three copies of the 7p22 genomic region that encodes miR-339 in GBM. The overall expression of this miRNA was found to be lower in gliomas than in the control brain tissue. It was therefore hypothesised that transcriptional or post-transcriptional events may impact the expression level of this particular miRNA.

### Expression control, post-transcriptional regulation and environmental effect

A high correlation in control brain tissues between the expression of miRNAs and their host genes was previously identified in miR-7-3, miR-9-1, miR-126, miR-128-1 and miR-139-5p [13]. We drew a similar conclusion for miR-7-3, miR-128-1, miR-128-2 and miR-139-5p but not for miR-9-1 and miR-126 in gliomas (Table 2). We can conclude that at least in miR-126, a co-regulated expression with EGFL7 appears to be disrupted in gliomas. A similar observation was also previously reported in the case of the human colon cancer [17].

Several miRNA coding regions are clustered in the genome. For example, miR-16, which was found to be over-expressed in both types of glioma, is encoded by the miR-16-1 gene that is clustered with miR-15a on chromosome 13q14.2 and by the miR-16-2 gene clustered with miR-15b on chromosome 3q25.33. Since we detected over-expression of miR-15b and no change in the miR-15a expression level in the two types of glioma, it can be concluded that only the miR-15b/miR-16-2 cluster is deregulated in these tumours. Another cluster, also dubbed oncomir-1, with the co-transcribed miR-17, miR-18, miR-19, miR-20a and miR-92 genes, is located on chromosome 13q31.3 [18]. It is over-expressed in gliomas, as shown by the high amounts of the mature forms of miR-17 and miR-20a. The absence of detection of the other members of the cluster might be the consequence of as yet not well defined post-transcriptional events. Stability of the precursors or mature miRNAs is probably controlled by processes that may affect the steady-state amounts of tumour miRNAs as well as the half-life of specific segments of the precursor transcripts (exemplified for miR-138 [19]). This also seems to hold true for miR-149, miR-330-3p and miR-339-5p, for example, since the amounts of these mature miRNAs were very low, whereas the expression level of the host gene transcript was nearly unaffected in gliomas compared to control brain tissue. All these observations suggest that the maturation process of miRNA transcripts and its deregulation remain to be investigated for all the individual miRNAs.

Epigenetic regulation of miRNA gene expression is also expected and has been reported to occur through methylation of CpG islands for miR-124 [10], miR-127 [11] and miR-128 [12], for example. In miR-124-2, miR-127, miR-128, miR-155 and miR-339 genes, we found that the CpG methylation levels in gliomas were nearly identical compared to control tissues. However, we found that upstream of the miR-210 and miR-330 genes the CpG methylation rates were higher in ODG than in GBM (Fig. 2). Higher expression of miR-210 in GBM than in ODG may therefore be related to the increased methylation rate for miR-210 in ODG, but a similar explanation for miR155 seems unlikely.

Finally, we found a number of genes that can be up-regulated in GBM cells *in vitro* by environmental conditions such as hypoxia. This is the case for miR-210, in agreement with previous studies that showed that miR-210 is over-expressed in low oxygen concentration in breast cancer or colon cancer cell lines [20], [21]. Interestingly, it can be hypothesised that in GBM, hypoxia may favour the increase of miR-210 concentration, whereas in ODG, excess promoter methylation may reduce expression of this miRNA.

### Tissue-specific miRNAs

miR-9, miR-124, miR-128, miR-132, miR-134 and miR-409-5p are known as brain-enriched or even brain-specific miRNAs (for review [22], [23]). In the rat, miR-9 is down-regulated during oligodendrocyte differentiation from oligodendrocyte progenitor cells [24]. A high amount of this miRNA in ODG might therefore be linked to a dedifferenciation process during tumorigenesis.

Tissue-specific expression of some miRNAs may lead to apparent over- or under-expression of these miRNAs in tumour tissues. For example, miR-124 is constitutively expressed in mature neurons [25]. It promotes neurite outgrowth during neuronal differentiation, possibly by regulation of the cytoskeleton [26]. miR-124 is the most abundant miRNA in the adult mammalian brain, accounting for 25%–48% of all brain miRNAs [27]. It was detected in lower amounts in anaplastic astrocytomas and glioblastomas than in non-neoplastic brain tissue [28]. miR-124 targets the mRNA that encodes PTBP1 (PTB/hnRNP I), a global repressor of alternative pre-mRNA splicing in non-neuronal cells [29].

miR-128 is more strongly expressed in neurons than in astrocytes [30]. miR-132 is enriched in neurons and is involved in neurite outgrowth [31]. Finally, miR-134 is present in dendrites, where it modulates the development of dendritic spines, neuronal protrusions that connect with other neurons and control neuronal transmission and plasticity [32], [33]. miR-134 and miR-409-5p genes are located in a very large cluster of brain-specific miRNAs at chromosome 14q32.31, as recently reported [23].

The low levels of miR-124, miR-128, miR-132, miR-134 and miR-409-5p observed in glioma tissues in this study therefore appears consistent with the fact that the tumour samples investigated were devoid of normal brain cellular components that express those miRNAs. There is general agreement that miR-128, miR-132 and miR-134 are detected in low amounts in gliomas (supplementary Table S5). It has also been reported that the members of the let-7 family, miR-21, miR-126, miR-221, and miR-222 are highly expressed in endothelial cells [34]. As a consequence, high levels of some of these miRNAs may be related to substantial vascularization in these tumours. However, in agreement with others [28], we did not find over-expression of mir-221 in gliomas.

### Functional rationale for miRNA deregulation in gliomas

The expected functional effects of a number of deregulated miRNAs in gliomas has been reviewed in the literature [4], [35]–[40]. Functional miRNAs have also been identified by integrative genome analysis or studies of the RISC-specific miRNA/mRNA profiles [41], [42]. However, a comprehensive understanding of the miRNA roles remains to be established. We described a non-exhaustive network that can illustrate the possible impacts on glioma cell pathways of some of the miRNAs found to be deregulated in this study (Fig. 3B). It is consistent that miR-124, miR-149 and miR-339-5p, three miRNAs that target PTBP1, a protein that was found in high amounts in gliomas, were down-regulated. PTBP1 has recently been reported to play a significant role in glioblastoma cell proliferation [43].

While this study was in progress, Malzkorn , *et al.* [44] also identified that the miR-17-92 cluster is over-expressed in glioblastomas. The miR-17-92 can down-regulate the expression of the proapoptotic protein Bim, an event that is related downstream to the over-expression of BCL2 [45]. Both the high levels of miR-17 and miR-20a and the low level of miR-139-5p that putatively target BCL2 are therefore consistent with the high level of BCL2 we observed in gliomas and are related to an expected antiapoptotic effect. miR-15b has also been reported to target BCL2 [46]. However, according to our results, the impact of the up-regulation of miR-15b on the expression of BCL2 seems to be less pronounced in gliomas. Antiangiogenic thrombospondin 1 (THBS1) is the target of miR-17-92 [47] and let-7f [34], which are all overexpressed in gliomas.

In addition, the expression of the miR-17-92 cluster was recently found to be positively controlled by STAT3 (signal transducer and activator of transcription 3) [48] and STAT3 expression is controlled by miR-17-92 [49]. Moreover, the expression of HIF-1α is presumed to be negatively controlled by miR17 / miR20a [50]. IL-6, whose production is stimulated by hypoxia and elevated in glioblastoma cells, stimulates the activation of the transcription factor STAT3. This is consistent with the increased amount of Y705-phosphorylated form of STAT3 observed in GBM. In glioblastomas, the higher the number of Y705P-STAT3-positive tumour cells, the poorer the outcome is, as reported by Birner , *et al.* [51]. Once activated, STAT3 favours the stabilisation of HIF-1α [52] that induces miR-210 overexpression [21]. In malignant human cholangiocytes, over-expression of IL-6 was described to up-regulate the expression of let-7-a, which in turn contributes to the increased phosphorylation of STAT3 [53]. This may be relevant to the well-known fact that elevated IL-6 ligand as well as receptor expression are associated with poor survival of patients with glioma [54].

STAT3 activation by acetylation is a process that requires CD44 and the active acetyl transferase (EP300: E1A binding protein p300) [16]. Since we evidenced in glioblastomas that the amounts of CD44 are high and that the amount of SIRT1 that inactivates EP300 is low, we can assume that this situation may facilitate activation of STAT3 by acetylation. All the above data make STAT3 an important factor in the physiopathology of glioma cells, in agreement with a recent report [55].

**Figure 4:**
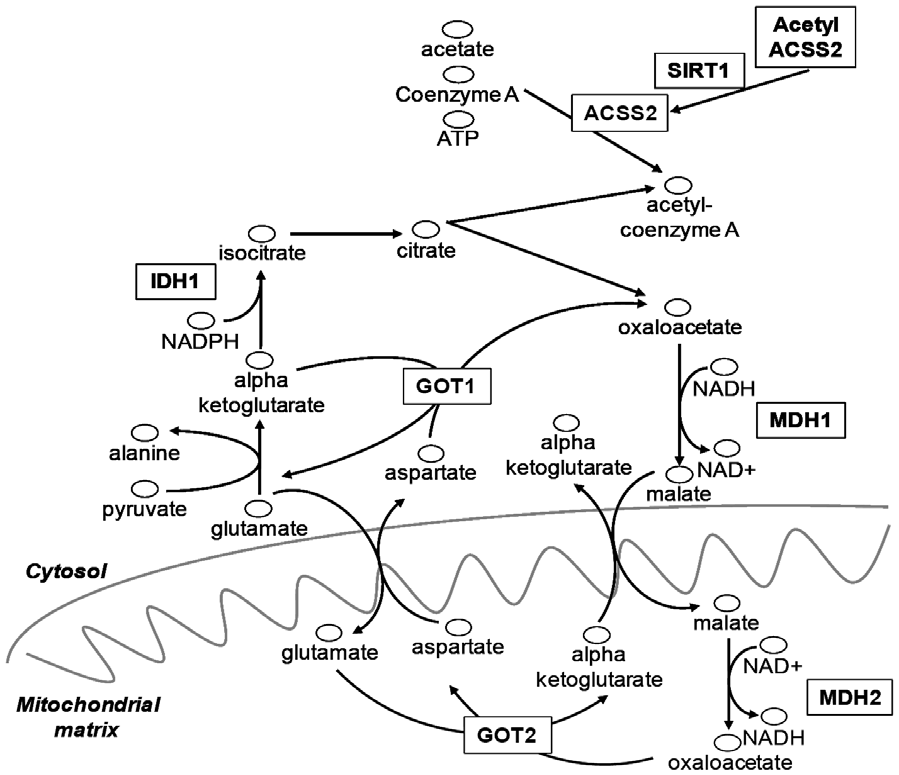
Metabolic pathways and enzymatic roles of MDH1, IDH1 and SIRT1. ACSS2: acyl-CoA synthetase short-chain family member 2; IDH1: isocitrate dehydrogenase 1 (NADP+), soluble1; GOT1: glutamic-oxaloacetic transaminase 1; GOT2: glutamic-oxaloacetic transaminase 2; MDH1: malate dehydrogenase 1, NAD (soluble); MDH2: malate dehydrogenase 2, NAD (mitochondrial); SIRT1: sirtuin 1. Inactivating mutations in IDH1 have been reported to be frequent in oligodendrogliomas [8].

In the cytosol, SIRT1 also deacetylates and activates acetyl-CoA synthetase (ACSS2) in the presence of oxidised NAD (Fig. 4). As a consequence of the absence of SIRT1 in gliomas, ACSS2-dependent production of acetyl-CoA from acetate will be aborted. Acetyl-CoA, an important metabolite for cell energy and a precursor at the cross-roads of several metabolic pathways, can also be produced, at least in adipose tissue, as an end-product of a pathway that starts by transamination between pyruvate and glutamate [56] (Fig. 4). In this pathway, IDH1, the cytosolic form of isocitrate dehydrogenase, produces isocitrate from alpha-ketoglutarate. In addition, oxaloacetate – a substrate of MDH1 – is co-produced with acetyl-CoA. If this glutamate-dependent acetyl-CoA generation pathway functions in glial cells, in gliomas the mutations of IDH1 or the low amounts of MDH1 are expected to reduce the concomitant production of acetyl-CoA and oxaloacetate through this pathway.

In fact, in oligodendrogliomas and secondary glioblastomas, mutations in the IDH1 gene that inactivate the IDH1 catalytic activity have been reported [8]. IDH1 mutations cause a gain of function, resulting in the production and accumulation of oncogenic 2-hydroxyglutarate [57]. In the present study, we evidenced that MDH1 was nearly undetectable by Western blot in our glioblastoma lysates. miR-15b, miR-16, miR-26b and miR-126, all four miRNAs that we found over-expressed in gliomas, putatively target MDH1 and may contribute to its down-regulation.

Mutations in IDH1 probably affect the cytosolic pool of alpha-ketoglutarate, one of the metabolites that fuels the mitochondrial aspartate-malate shuttle (Fig. 4). The malate-aspartate shuttle allows cytosolic oxidation of NADH and concomitant reduction of mitochondrial NADH. MDH1 is the extra-mitochondrial form of malate dehydrogenase and plays a crucial role in the malate-aspartate shuttle. The absence of MDH1 prevents this shuttle from functioning. Interestingly, another critical impact on the mitochondrial oxidative phosphorylation system resulting from the repression of the iron-sulfur cluster assembly proteins by a high level of miR-210 was recently reported [58]. The control of the energy metabolism in tumours by miRNAs may contribute to the aggressive phenotypes of the tumours [59], [60].

In addition, in a normal cell context, it was described that nuclear translocation and activation of p53 are promoted by MDH1 and hampered by MDM2 [61]. Thus, both the absence of MDH1 and the over-expression of MDM2, which we showed in glioblastomas, may negatively control p53 activation and then enhance tumor formation and growth.

In conclusion, modified amounts of active SIRT1, IDH1 and MDH1 in gliomas may interfere with acetyl-CoA production. This may have important consequences that have yet to be understood on energy production and anabolism in gliomas. The absence of MDH1 in primary glioblastomas may dramatically impair the mitochondrial NAD reduction through the aspartate-malate shuttle.

Finally, it can be concluded that current limited knowledge on miRNA targets makes it difficult to anticipate the impact of miRNA deregulations in tumour tissues. Large-scale analysis as initiated by others [62]–[64] is required to obtain more details on the intricate networks between mRNA, miRNA and proteins.

## 

## Supporting Information

Figure S1 miRNA gene copy numbers in gliomas. Genomic DNA from cells or tissues was extracted using standard protocols (Qiagen) and was used to assess gene copy number variations between tissues by three different technical approaches. *Method 1:* PCR products obtained with primers listed in supplementary Table S4 and with two different amounts of purified DNAs for each sample were run in agarose gels, stained by ethidium bromide and pictured with the ChemiDoc system and Image Lab software (BioRad). All signal intensities were normalised with beta actin PCR product amplified from the same samples. The ratios between the normalised amount of a gene fragment in gliomas and the amount of the equivalent fragment in control samples were calculated. *Method 2:* real-time PCR with SYBR-green was used to quantify gene copy number of mir-9-3, miR-124-1. Four different amounts of DNA were used for each sample. *Method 3:* hybridisation on Affymetrix SNP-6.0 arrays. Gene copy number was analysed in glioblastoma low-passage cell lines using Affymetrix SNP-6.0 arrays (according to the manufacturer’s instructions). The quality control assessment for the SNP-6.0 arrays was performed using three parameters: the Contrast Quality Control (CQC) greater than 0.4, the call rate control over 86% and the Median of the Absolute values of all Pairwise Differences (MAPD) less than 0.4. Virtual karyotypes were generated using Affymetrix Genotyping Console software 3.0 using the reference file created from HapMap samples. This approach only revealed a modified copy number in the miR-339 coding region (7p22.3 fragment bp 52,899 to 1,466,894). The ratios between the normalised amount of a gene fragment and the amount of the equivalent fragment in control samples were calculated and reported in the figure. Black bars: GBM/N ratios; white bars: ODG/N ratios.
(TIF)

Figure S2 Effect of oxygen concentration on miRNA and gene expressions in cultured U87 glioblastoma cells. Cultured cells were submitted to different oxygen concentrations (20%, 3% and 0.3%). *A,* Cellular miRNA contents of miR-100 (diamonds) and miR-210 (squares) were assessed by RT-qPCR. *B,* mRNA expression levels of carbonic anhydrase IX (CA9), hypoxia-inducible gene-2 (HIG2), lysyl oxidase (LOX) and GAPDH were measured by hybridisation to GeneChip^®^ Human Genome U133 Plus 2.0 (Affymetrix) at three oxygen concentrations: 20% (white bars), 3% (grey bars) or 0.3% (black bars). All data are reported in arbitrary units; y-axes are in logarithmic scales.
(TIF)

Figure S3 Full length blot for detection of MDH1 in glioma samples. Lanes 1 and 2: control; Lanes 3 and 4: ODG; lanes 5 and 6: GBM. Molecular masses in kDa. Experimental conditions as reported in Materials and Methods and Fig. 3.
(TIF)

Figure S4 Immunodetection of STAT3 and tyrosine705-phosphorylated STAT3 in control and tumour samples. Experimental conditions as reported in Materials and Methods and Fig. 3. Tissue lysates from control brain samples and tumour samples (ODG and GBM).
(TIF)

Table S1 Classification of the analysed tumour samples according to clinical and analytical parameters. a) Tumours were classified according to the WHO classification. b) Tumours were typed based on transcriptomic analyses. mRNA profiles were compared to data published by Li , *et al.* [3]. Tumours were classified either as group O (corresponding to short survival patients) or G (corresponding to long-survival patients) according to the coefficient of variation (indicated as percentage) with the published corresponding tumour group signatures. c) Tumour typing was based on transcriptomic analyses. mRNA profiles were compared to data published by Phillips , *et al.* [2]. Tumours were classified either as PN (proneural group, corresponding to low-grade tumours) or MES (mesenchymatous group, corresponding to high-grade tumours) according to the highest coefficient of variation with the published tumour group signatures. d) The mutation in codon 132 of IDH1 was analysed by sequencing the corresponding amplified DNA fragment [8]. WT, wild type. e) Losses of heterozygosity (LOH) at loci 1p and 19q were analysed by FISH or MLPA [65]. f) Scoring was calculated on the following basis: A value of 0 was noted for the following results: ODGII, O, PN, mutated IDH1, LOH, No hypersignal with gadolinium, positive response to treatment, and survival period longer than 36 months, for parameters 1–8 respectively. On the contrary, a value of 10 was scored for the following results: GBM, ODGIII or OAII for parameter 1, or G, MES, WT IDH1, no LOH, hypersignal with gadolinium, no response to treatment, survival period shorter than 36 months, for parameters 2–8 respectively. The final scoring was assessed using the equation: ((*a*×0)+(*b*×10))/(*a*+*b*), where *a* is the total number of parameters with a value of 0 and *b* is the total number of parameters with a value of 10 for each sample. Ideally, low grade tumours should be characterised by a score of 0 and high grade tumours by a score of 10.
(XLS)

Table S2 PCR and pyrosequencing primers for DNA methylation analysis. F: Forward primer; R: Reverse primer; S: sequencing primer. * 5’ end biotinylated primers. † TSS1, TSS2: transcription start site 1 and 2, respectively.
(XLS)

Table S3 List of the miRNAs assayed by membrane-array hybridization and real-time PCR. For miRNA real-time PCR analysis, LMW RNAs (80 ng) were reverse-transcribed with a Taqman^®^ MicroRNA Reverse Transcriptase kit (Applied Biosystems) and Taqman^®^ microRNA assay containing gene-specific stem-loop primers. Then the real-time PCR is performed using Taqman^®^ PCR Universal Master Mix, No AmpErase^®^ UNG (Applied Biosystems) and the Stratagene Mx3005 (Stratagene Inc.). Five small nucleolar RNAs (RNU6B, RNU19, RNU24, RNU44 and RNU66) were also assayed by real-time PCR and RNU24 was determined as the best normaliser by using the Normfinder algorithm [66]. Individual values were normalised with the value of RNU24 using Relative Expression Software Tool (REST) [67].
(XLS)

Table S4 miRNA PCR primers for gene copy number analysis. F: Forward primer; R: Reverse primer.
(XLS)

Table S5 microRNA expression in gliomas. Data from the literature. Numbers refer to references indicated below. 1. Ciafre SA, Galardi S, Mangiola A, Ferracin M, Liu CG , *et al.* (2005) Extensive modulation of a set of microRNAs in primary glioblastoma. Biochem Biophys Res Commun 334 (4): 1351–1358. 2. Conti A, Aguennouz M, La Torre D, Tomasello C, Cardali S , *et al.* (2009) miR-21 and 221 upregulation and miR-181b downregulation in human grade II-IV astrocytic tumors. J Neurooncol 93(3): 325–332. 3. Gaur A, Jewell DA, Liang Y, Ridzon D, Moore JH , *et al.* (2007) Characterization of microRNA expression levels and their biological correlates in human cancer cell lines. Cancer Res 67(6): 2456–2468. 4. Godlewski J, Nowicki MO, Bronisz A, Williams S, Otsuki A , *et al.* (2008) Targeting of the Bmi-1 oncogene/stem cell renewal factor by microRNA-128 inhibits glioma proliferation and self-renewal. Cancer Res 68(22): 9125–9130. 5. Landgraf P, Rusu M, Sheridan R, Sewer A, Iovino N , *et al*. (2007) A mammalian microRNA expression atlas based on small RNA library sequencing. Cell 129(7): 1401–1414. 6. Lavon I, Zrihan D, Granit A, Einstein O, Fainstein N , *et al*. (2010) Gliomas display a microRNA expression profile reminiscent of neural precursor cells. Neuro Oncol 12(5): 422–433. 7. Malzkorn B, Wolter M, Liesenberg F, Grzendowski M, Stuhler K , *et al*. (2009) Identification and Functional Characterization of microRNAs Involved in the Malignant Progression of Gliomas. Brain Pathol 20(3): 539–550. 8. Nelson PT, Baldwin DA, Kloosterman WP, Kauppinen S, Plasterk RH , *et al*. (2006) RAKE and LNA-ISH reveal microRNA expression and localization in archival human brain. RNA 12(2): 187–191. 9. Ridzon D, Tan R, Nguyen J, Broomer A, Caifu C. (2005) MicroRNA Expression Signature in Human Glioblastoma Multiforme Brain Tumor. http://www3.appliedbiosystems.com/cms/groups/mcb_marketing/documents/generaldocuments/cms_042004.pdf. 10. Silber J, Lim DA, Petritsch C, Persson AI, Maunakea AK , *et al*. (2008) miR-124 and miR-137 inhibit proliferation of glioblastoma multiforme cells and induce differentiation of brain tumor stem cells. BMC Med 6: 14. 11. Webster RJ, Giles KM, Price KJ, Zhang PM, Mattick JS , *et al.* (2009) Regulation of epidermal growth factor receptor signaling in human cancer cells by microRNA-7. J Biol Chem 284(9): 5731–5741. Ref 11: data for ODG.
(XLS)
