# Supplementary figures and images for: MicroRNA and Target Protein Patterns Reveal Physiopathological Features of Glioma Subtypes
Source: PLoS One. 2011 May 31;6(5):e20600. doi: 10.1371/journal.pone.0020600 (PMC3105101; doi:10.1371/journal.pone.0020600)

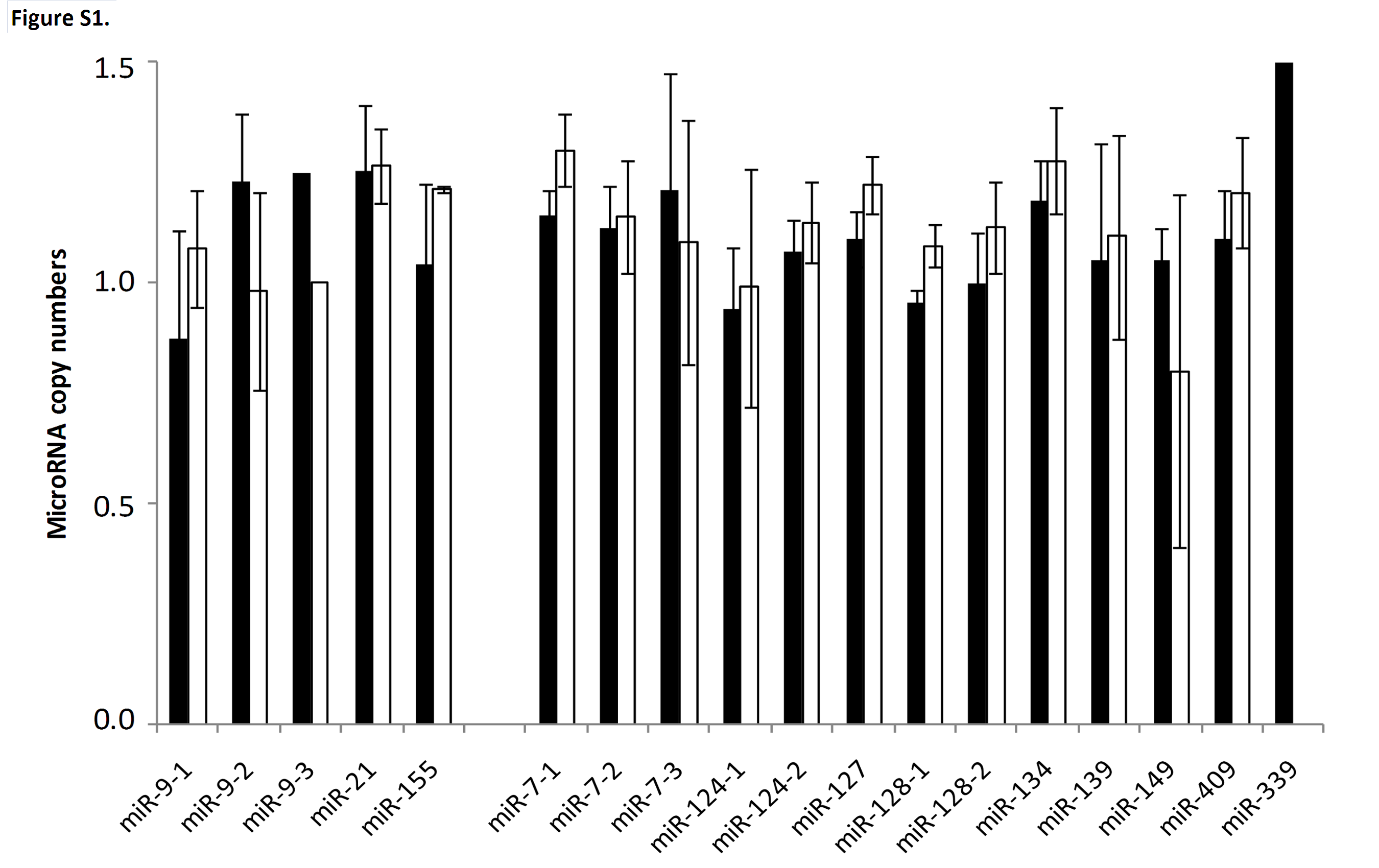

Supplement: Figure S1 — miRNA gene copy numbers in gliomas. Genomic DNA from cells or tissues was extracted using standard protocols (Qiagen) and was used to assess gene copy number variations between tissues by three different technical approaches. Method 1: PCR products obtained with primers listed in supplementary Table S4 and with two different amounts of purified DNAs for each sample were run in agarose gels, stained by ethidium bromide and pictured with the ChemiDoc system and Image Lab software (BioRad). All signal intensities were normalised with beta actin PCR product amplified from the same samples. The ratios between the normalised amount of a gene fragment in gliomas and the amount of the equivalent fragment in control samples were calculated. Method 2: real-time PCR with SYBR-green was used to quantify gene copy number of mir-9-3, miR-124-1. Four different amounts of DNA were used for each sample. Method 3: hybridisation on Affymetrix SNP-6.0 arrays. Gene copy number was analysed in glioblastoma low-passage cell lines using Affymetrix SNP-6.0 arrays (according to the manufacturer’s instructions). The quality control assessment for the SNP-6.0 arrays was performed using three parameters: the Contrast Quality Control (CQC) greater than 0.4, the call rate control over 86% and the Median of the Absolute values of all Pairwise Differences (MAPD) less than 0.4. Virtual karyotypes were generated using Affymetrix Genotyping Console software 3.0 using the reference file created from HapMap samples. This approach only revealed a modified copy number in the miR-339 coding region (7p22.3 fragment bp 52,899 to 1,466,894). The ratios between the normalised amount of a gene fragment and the amount of the equivalent fragment in control samples were calculated and reported in the figure. Black bars: GBM/N ratios; white bars: ODG/N ratios. (TIF) [file pone.0020600.s001.tif]

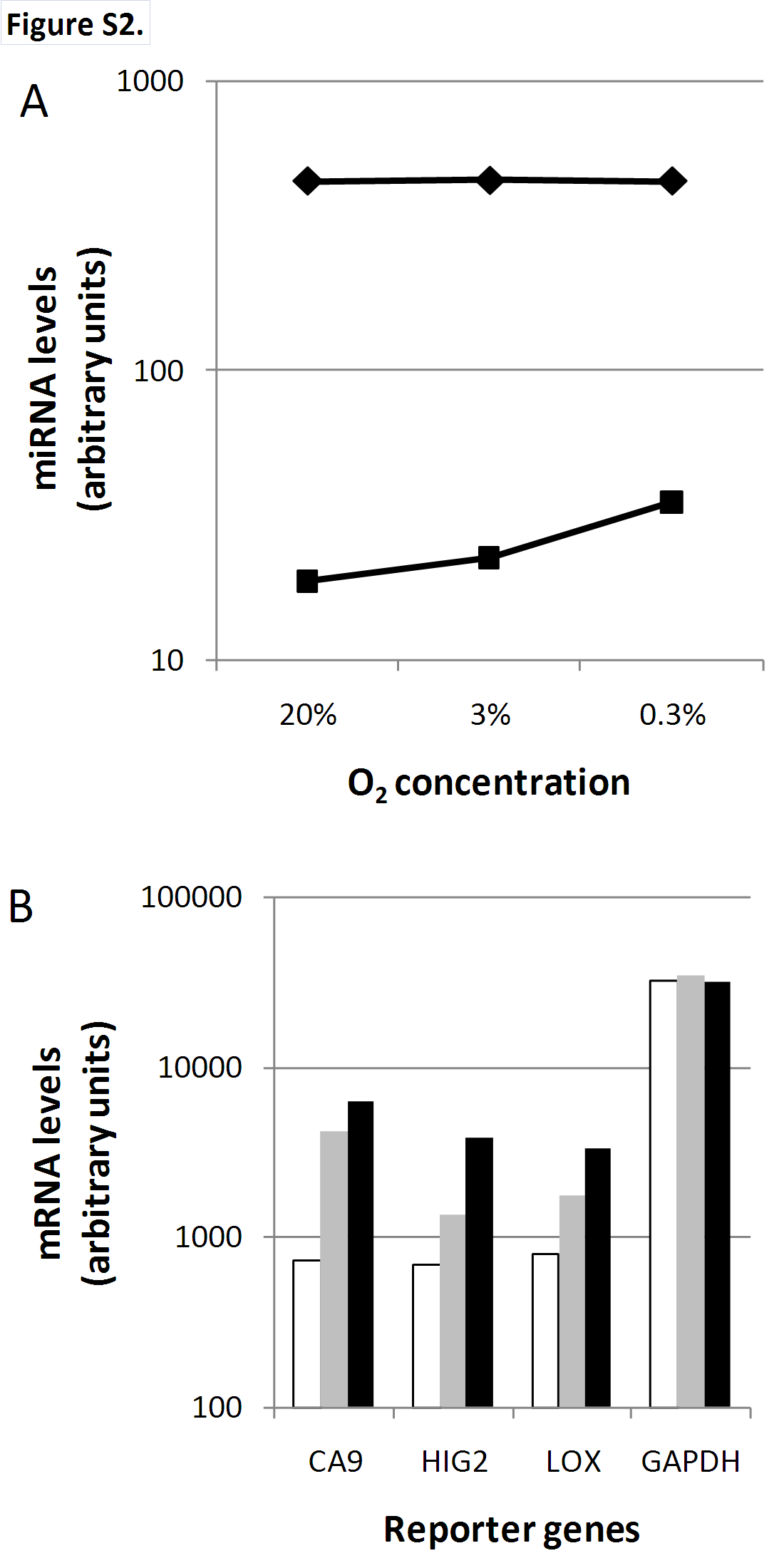

Supplement: Figure S2 — Effect of oxygen concentration on miRNA and gene expressions in cultured U87 glioblastoma cells. Cultured cells were submitted to different oxygen concentrations (20%, 3% and 0.3%). A, Cellular miRNA contents of miR-100 (diamonds) and miR-210 (squares) were assessed by RT-qPCR. B, mRNA expression levels of carbonic anhydrase IX (CA9), hypoxia-inducible gene-2 (HIG2), lysyl oxidase (LOX) and GAPDH were measured by hybridisation to GeneChip® Human Genome U133 Plus 2.0 (Affymetrix) at three oxygen concentrations: 20% (white bars), 3% (grey bars) or 0.3% (black bars). All data are reported in arbitrary units; y-axes are in logarithmic scales. (TIF) [file pone.0020600.s002.tif]

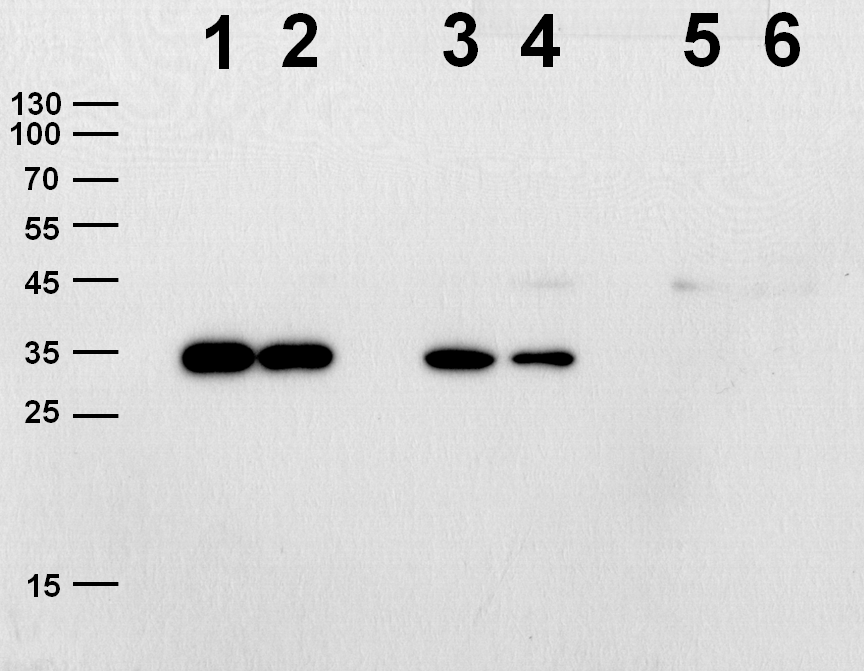

Supplement: Figure S3 — Full length blot for detection of MDH1 in glioma samples. Lanes 1 and 2: control; Lanes 3 and 4: ODG; lanes 5 and 6: GBM. Molecular masses in kDa. Experimental conditions as reported in Materials and Methods and Fig. 3. (TIF) [file pone.0020600.s003.tif]

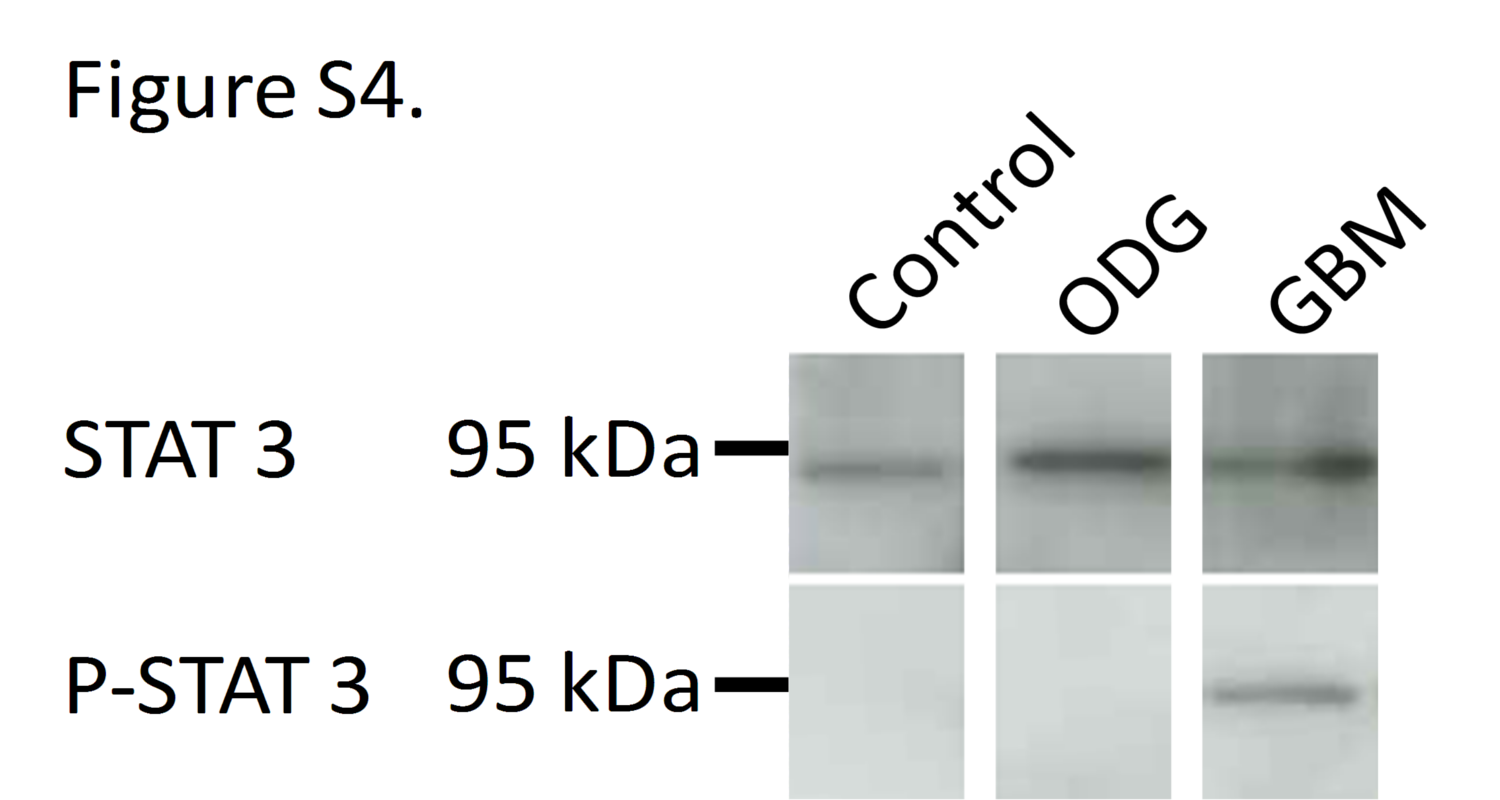

Supplement: Figure S4 — Immunodetection of STAT3 and tyrosine705-phosphorylated STAT3 in control and tumour samples. Experimental conditions as reported in Materials and Methods and Fig. 3. Tissue lysates from control brain samples and tumour samples (ODG and GBM). (TIF) [file pone.0020600.s004.tif]
